# Factors Associated with Levels of Latrine Completion and Consequent Latrine Use in Northern Ghana

**DOI:** 10.3390/ijerph16060920

**Published:** 2019-03-14

**Authors:** Abraham Marshall Nunbogu, Miriam Harter, Hans-Joachim Mosler

**Affiliations:** 1 Department of Environmental and Social Science Research, Swiss Federal Institute of Aquatic Science and Technology, Urberlandstrasse 133, 8600 Dubendorf, Switzerland; miriam.harter@eawag.ch (M.H.); hans-joachim.mosler@eawag.ch (H.-J.M.); 2 Department of Geography and Environmental Management, University of Waterloo, 200 University Avenue West, Waterloo, ON N2L 3G1, Canada

**Keywords:** open defecation, latrine completion, latrine use, behaviour change, RANAS, CLTS, Ghana

## Abstract

Open defecation is still a major health problem in developing countries. While enormous empirical research exists on latrine coverage, little is known about households’ latrine construction and usage behaviours. Using field observation and survey data collected from 1523 households in 132 communities in northern Ghana after 16 months of implementation of Community Led Total Sanitation (CLTS), this paper assessed the factors associated with latrine completion and latrine use. The survey tool was structured to conform to the Risk, Attitude, Norms, Ability and Self-regulation (RANAS) model. In the analysis, we classified households into three based on their latrine completion level, and conducted descriptive statistics for statistical correlation in level of latrine construction and latrine use behaviour. The findings suggest that open defecation among households reduces as latrine construction approaches completion. Although the study did not find socio-demographic differences of household to be significantly associated with level of latrine completion, we found that social context is a significant determinant of households’ latrine completion decisions. The study therefore emphasises the need for continuous sensitisation and social marketing to ensure latrine completion by households at lower levels of construction, and the sustained use of latrines by households.

## 1. Introduction

According to the Joint Monitoring Program (JMP) on Water, Sanitation and Hygiene [1], approximately 844 million people lacked basic drinking water and 2.3 billion were without basic sanitation in 2015 with significant disparities across regions. Although sub-Saharan Africa (SSA) made modest progress in water and sanitation access between 1990 and 2015, 72% of its population are without basic sanitation at the end of the Millennium Development Goals (MDG) period [1]. Poor sanitation has overwhelmingly been approached as a health concern related to the consumption of water and food contaminated with fecal bacteria. Diarrhoeal disease accounts for about 8.6% of under-five mortality worldwide [2], and inadequate water and sanitation have been associated with diarrhoea [3,4].

For decades, sanitation approaches adopted by governmental and non-governmental organizations assessed the sanitation needs of households and provided either free or subsidized latrines [5]. These approaches did not address the issues of sanitation behaviour change and community empowerment that could promote sustained access to improved sanitation [6,7]. Following these results, Community-Led Total Sanitation (CLTS) was adopted. This approach focuses on behavioural change to create “open defecation free (ODF) communities” [8]. CLTS approaches open defecation as a collective health hazard, triggers communities for change, and inspires local innovation and mutual support for latrine construction and latrine use. 

Although CLTS has significantly facilitated the process of stopping open defecation [9], the 75% latrine coverage threshold required for improved health has not yet been reached [10,11,12], and problems persist with the long-term sustainability of latrines [13]. In general, research has shown that the adoption and sustained use of latrines is largely rooted in the social norms and networks of communities [14,15] and the rate of adoption of sanitation behaviours varies amongst households [16]. Therefore, households’ decision to construct and use a latrine may be influenced by the social norms of the community. While research on CLTS in developing countries has been extensive [10,13,16], few studies examine why people change their behaviour from a psychosocial perspective. However, understanding the mechanisms underlying behaviour change interventions is germane to improving its effectiveness [17]. 

To understand latrine completion and use decisions among households, we used the Risks, Attitudes, Norms, Abilities, and Self-regulation (RANAS) model of behaviour change [18] as a conceptual guide. The RANAS model underscores the importance of psychosocial factors in determining households’ sanitation behaviours [13,19,20,21,22]. The risk factors represent a person’s understanding of the health risk of open defecation, the person’s perceived perception of his or her risk of contracting diarrhoea, and his or her perception of the severity of diarrhoea and its consequences. The attitude factors are a person’s beliefs about the costs (money and time/effort) and benefits (good health, greater respect, personal safety) of constructing a latrine and his or her positive feelings about owning a latrine. Norm factors represent the perceived social pressure to construct a latrine. They describe a person’s observation and awareness of others’ behaviour, his or her perception as to which behaviours are typically practised. Norm factors also describe a person’s perception as to which behaviours are typically practised approved or disapproved by relatives, friends, or neighbours. This includes the awareness of the dos and don’ts expressed by a village, tribe, or religious leaders and other institutions. The ability factors take three forms: confidence in performance (a person’s perceived ability to organize and execute latrine construction), confidence in continuation (a person’s perceived ability to continue latrine construction and ability in being able to deal with barriers that arise), and confidence in recovering (a person’s perceived ability to recover from setbacks, to continue the construction and after disruptions). Self-regulation factors denote a person’s strategies for constructing a latrine; his or her plans for overcoming potential barriers in the course of latrine construction; and monitoring to keep construction on track. 

The psychosocial factors are embedded in contextual landscapes that may also influence latrine construction decisions. Psychosocial characteristics include social, physical, and personal factors. The social context represents the cultural and social relations, policies and laws, economic conditions (household’s income), and the information environment (including sanitation campaigns such as CLTS). Jenkins and Scott [23] argue that the cost of household latrines is a significant constraint on latrine construction. Yet, researchers [24,25] have stated that the policies and legislation on sanitation are least accessible to marginalized groups in ways that limit their ability to switch to latrine use. In CLTS, social relations and cohesion among community members are key for collective actions and mutual support in latrine construction [20]. 

The physical context includes the natural and built environment. For instance, whether the soil is loose or firm has to be considered when choosing the best fit design for latrine substructure so as to prevent latrine collapse. Finally, the personal context is framed by socio-demographic factors such as age, sex, religion, and education. Some studies [26,27,28] have shown that latrine construction and use is associated with households’ educational status, though Oljira and Berkessa [29] proposed a contrary view. 

In a study commissioned by the World Health Organisation (WHO), Garn et al. [11] reported that latrine coverage and ownership do not necessarily translate into latrine use. Even among households with latrines, open defecation is often still practised [30,31]. However, little is known about the households that have not been able to complete the construction of their latrines during CLTS interventions. In addition, the existing literature has not examined how level of latrine completion is associated with defecation habits among households. Achieving the Sustainable Development Goals (SDGs) is likely to be aided by better understanding of the factors associated with levels of latrine completion and consequent latrine use. This can inform effective targeting of interventions to increase sanitation coverage and subsequently improve health. Consequently, this study addresses the following questions:

(1) How do the levels of latrine completion relate to latrine use?
(2) How are socio-demographic characteristics of households linked to levels of latrine completion?
(3) What factors may deter households of different latrine completion levels from using their latrines?
(4) How do psychosocial factors differ between households’ latrine completion levels?
(5) How do reported reasons for latrine building differ between households’ latrine completion levels?

## 2. Methods

### 2.1. Research Context and Sampling 

This study was conducted in two neighbouring districts: Bole district and Sawla-Tuna-Kalba district in the Northern Region of Ghana. The Ghana Multi Indicator Cluster Survey (MICS) conducted in 2012 [32] revealed that about 71.9% of residents in the Northern Region practiced open defecation. At the district levels, similar results were observed with about 69.2% and 91.6% of the population defecating openly in Bole and Sawla-Tuna-Kalba Districts, respectively [33]. To address the sanitation menace, Global Communities, a Non-Governmental Organisation in collaboration with the local governments of the two districts implemented CLTS in the last quarter of 2016. Baseline data was collected prior to the intervention and a follow-up survey was conducted 4–6 months after the intervention was implemented. End-line data for the intervention was collected 14–16 months after the intervention from February to March, 2018. During the baseline, 25 households were randomly selected from each community following Hoffmeyer-Zlotnik [34] random route method. Equal chances were given to both men and women to participate in the survey, because both might be involved in decisions concerning latrine construction. Respondents for the baseline survey were interviewed again in the follow-up surveys. Every participant gave informed written consent to participate in the interviews. The ethical board of the University of Zurich, Switzerland and the Ethical Review Committee of the Ghana Health Service (GHS-ERC: 05/01/2016) approved this research trial. 

### 2.2. Data Collection 

A team of three supervisors and 33 local data collectors were recruited for data collection. The unit of study was the household, defined in this context as a person or group of people who live together in a dwelling and share housekeeping and cooking arrangements. Before data collection, one week of training was organized for the three field supervisors. After training the field supervisors, the data collectors were trained for another week. The training included discussing and interpreting and translating the survey questions into the various local languages (Brefo, Dagare, Gonja, Safalba, Twi, Mo and Waali) spoken in the study area to ensure the team understood the questions and the study. During the training, data collectors were grouped by language to rehearse the questions and to help each language group to adopt uniform words and terminologies. This was followed by role-plays at both the language group and general group levels to test data collectors’ interview and communication skills. The questionnaire was pretested in two days in four communities, and debriefing took place after every day of the pretest to share field experiences and adapt the instrument as necessary. The survey and questionnaire asked about socio-demographic characteristics, open-defecation habits, latrine construction and latrine use, psychosocial determinants of latrine construction and latrine use of households, and the physical and social context of the communities. The survey included some observations, which were recorded by the data collectors based on their individual judgement and joint decisions taken during training. The research manager and the field supervisors monitored the data collectors closely when the questionnaire was administered. Every data collector was assigned five respondents daily, and the research manager crosschecked the interviews for data quality after every day of data collection. 

### 2.3. Measures

#### 2.3.1. Latrine Construction

Latrine construction was derived from the question ‘Does your household have its own latrine?’ and coded as 0 = no household latrine; and 1 = household latrine. Since our focus is on households latrine construction levels and corresponding use, we dropped those with no household latrine. This resulted in an analytical sample of 1523 households. 

#### 2.3.2. Latrine Usage

Latrine use of respondents was measured by a series of questions that sought to assess respondents’ defecation habits in the morning, midday, and evening of the 7 days prior to the interview. For open defecation, the question ‘how many of the last 7 mornings did you defecate in the open?’ was asked. Respondents were also asked ‘how many of the last 7 mornings did you defecate in the latrine?’ to assess their latrine use in the previous 7 days. Based on the responses given, data collectors were asked to decide whether to code the respondent 1 = use latrine exclusively, 2 = defecate in the open exclusively and 3 = use latrine and does open defecation. For this analysis we added the mixed users to the open defecation group.

#### 2.3.3. Socio-Demographic Factors

Socio-economic and demographic factors included in the study were individual monthly income, highest formal education attainment, marital status and age. Household characteristics included household size, number of children, number of children under 5 years, and number of adult men and women (above 17 years).

#### 2.3.4. Psychosocial Determinants

These were assessed using the RANAS approach [12,18]. All questions were answered on a 5-point Likert scale. We used a visual scale of 5 black points of varying sizes to guide respondents select one of answering options. Data collectors read out every option to the respondent while indicating it on the visual scale. 

#### 2.3.5. Level of Latrine Construction

Level of latrine construction was reported by respondents and physically verified by data collectors. We categorised construction into five levels. Level 1: Only pit is dug; Level 2: Pit is dug and superstructure constructed; Level 3: Pit is dug, superstructure constructed, and latrine is roofed; Level 4: Pit is dug, superstructure constructed, latrine is roofed and has door; and Level 5: Pit is dug, superstructure constructed, latrines is roofed, and latrine has door and a vent pipe. These levels are described in the results. 

### 2.4. Data Analysis Procedure 

To answer the first research question, we classified households into five groups based on the stages of their latrines construction as described in the measures above. After this classification, we used Chi-square tests to determine the statistical difference in the latrine use and open defecation habits between the five levels. Using the results of the Chi-square test, we re-grouped the households into three levels based on the probable influencers of latrine use. These three levels are: (1) households that only dug the pit and constructed superstructure; (2) households that dug the pit, constructed superstructure, and roofed the latrine; and (3) households that dug the pit, constructed superstructure, roofed latrine, and fitted door and vent pipes. Two levels were compared at a time (Level 1 vs. Level 2 and Level 2 vs. Level 3). 

To answer the remaining research questions, we again compared the three levels of latrine completion progressively. An ANOVA was used to test the socio-demographic factors, psychosocial factors, and other factors that may be linked to latrine construction and use between the three levels. For multiple response items, we did not test the statistical significance between the percentages but reported them relatively. All data analysis was carried out using SPSS version 22 (IBM SPSS Statistics for Windows, Version 22.0. IBM Corp, Armonk, NY, USA).

## 3. Results

The sample included 65.1% male respondents with mean age as 45 years (Standard Deviation: (SD) = 16.1). Of all the respondents, 84.8% reported they were married. On the average, respondents had attended school for 1.9 years (SD = 3.90), 15% were able to read or write. Approximately, 53.1% were Christians, 13.3% were Muslims, and 32.6% affiliated with Traditional and other religious groups. Agriculture was the main economic activity, with about 89.9% of respondents engaged in that sector. The average household size was nine members (SD = 5.95), with an average of two children (SD = 1.97) below the age of 5 years per household. The mean monthly income was 156.3 Ghana Cedis (4.7 Ghana Cedis = 1 US Dollar; exchange rate 26 September 2018) per household (SD = 53.88). Soil conditions varied among the study communities and households. Majority of households (55.4%) described their soil as a mixture of loam, sand, clay, and gravel. To answer the first research question (How do levels of latrine completion relate to latrine use?) we classified the households into five levels based on the construction stage of their latrine. 

### 3.1. Level 1 (L1): Only Pit Is Dug

**Figure 1:**
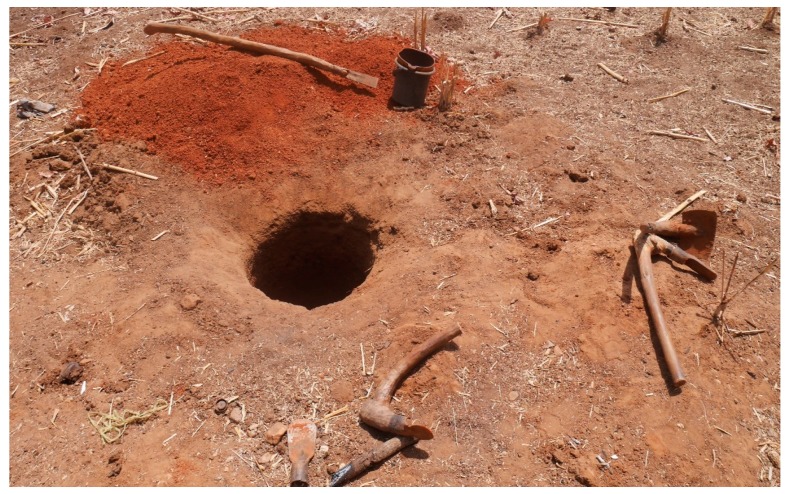
Only pit is dug.

Digging a pit is usually the first stage of latrine construction (see Figure 1). This stage often precedes securing of site for latrine construction from household members or sometimes from community leaders. For many rural communities in Ghana, this involves a lot of physical work, since rudimentary tools such as shovel and pick axe are mostly used in digging. 

Respondents mentioned that this stage of latrine construction is not usually performed by women because it involves rough and physical work. Therefore, in female-headed households, pits are dug by their male children, relatives living in and around their community, or by hired labour. Households are usually advised to excavate the pit to a depth of 2 m, depending on the soil condition. The pit is often unlined; thus allowing the fecal liquid to drain into the soil and leaving sludge behind.

### 3.2. Level 2 (L2): Pit Is Dug and Superstructure Constructed (Pit + Superstructure)

**Figure 2:**
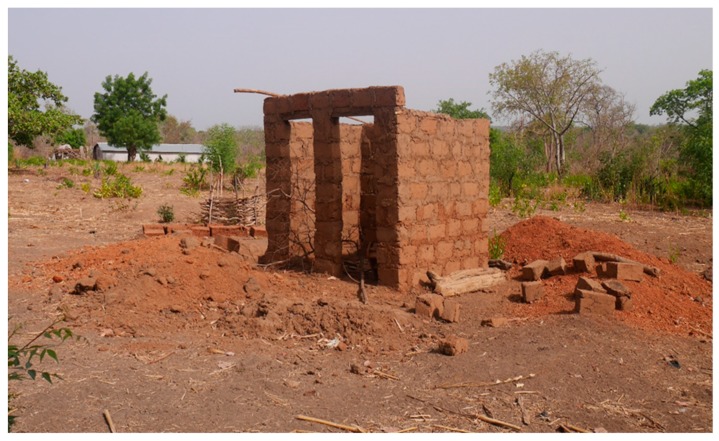
Pit is dug and superstructure constructed.

After excavating the pit, it is decked and the superstructure constructed as illustrated in Figure 2. The covering slab is usually made of wood and earth. During decking, the drop hole is created and provisions are made for the vent pipe if the household has the intention of installing one. The superstructure is built of locally available materials. Field observations revealed that 60.3% of the superstructures were made of mud, and 21.6% from bricks and other materials. The type of material used for constructing the superstructure depends on the household’s financial capacity, the availability of construction material locally, cultural and local architecture, and the quality of artisans available in the community.

### 3.3. Level 3 (L3): Pit Is Dug, Superstructure Constructed and Latrine Is Roofed (Pit + Superstructure + Roof)

**Figure 3:**
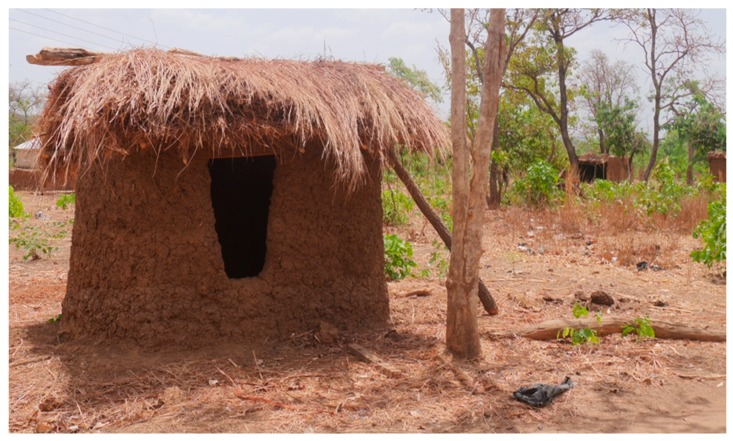
Pit is dug, superstructure constructed and latrine is roofed.

The third stage involves roofing the superstructure as shown in Figure 3. The main function of the roof is to prevent water from entering the privy room and the pit when it rains. It also provides shade from sunrays. In rural Ghana, latrine roofs are generally made of wattle and daub, thatch, and iron sheets in some households. We found that 63.6% of latrine superstructures were roofed with iron sheets and wood, 19.4% with mud only, 8.6% with mud and wood, and about 4.3% with thatched. Latrine construction in the study communities is gendered. While women fetch water and sand, men engage in the construction. However, where men are not available, women are involved in the raising of the superstructure. In addition, it is mostly women who plaster the walls, add the roof, and construct the squat plate when local raw materials are used. The walls are plastered with a local mortar made of earth, cow dung, ash, and water. 

### 3.4. Level 4 (L4): Pit Is Dug, Superstructure Constructed, Latrine Is Roofed and Has Door (Pit + Superstructure + Roof + Door)

**Figure 4:**
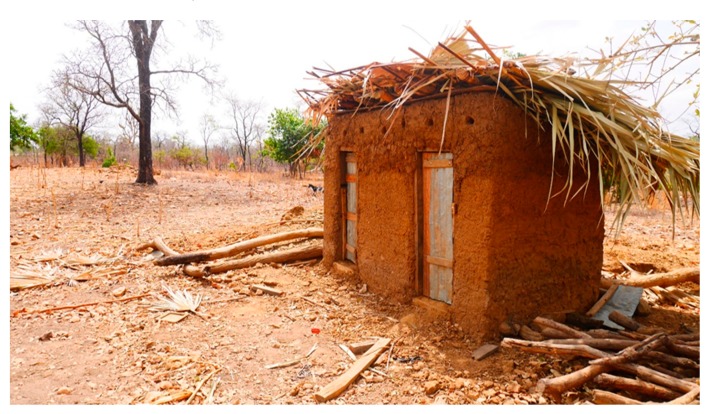
Pit is dug, superstructure constructed, latrine is roofed and door is fitted.

Level 4 comprises latrines which protect the privacy of users (see Figure 4). Privacy is guaranteed when a latrine door can be completely shut when in use. There are various types of latrine doors, and type used by households depends on preference, financial capacity, and the availability of construction materials. Approximately 12.5% of households covered the entrance to their latrines with mat, used cloth, and thatch, 16.5% constructed the doors with wood and iron sheets, 17.1% used metal, while 42.1% used only wood. 

### 3.5. Level 5: Pit Is Dug, Superstructure Constructed, Latrines Is Roofed, Latrine Has Door and a Vent Pipe (Pit + Superstructure + Roof +Door + Vent Pipe)

**Figure 5:**
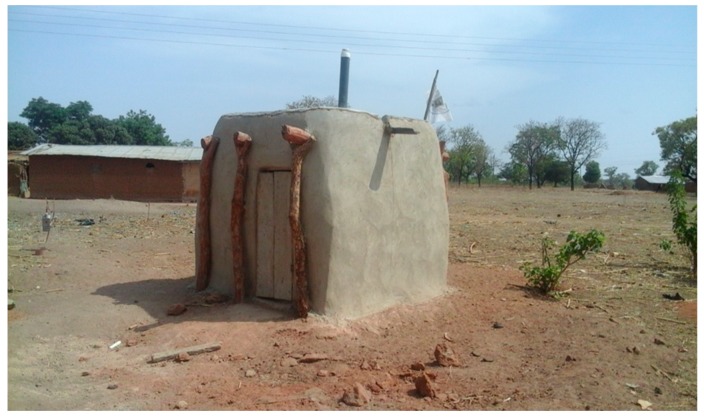
Pit is dug, superstructure constructed, latrines is roofed, latrine has door and a ventpipe.

Level 5 consists of latrines with vent pipes. The vent pipe is an improved feature of the latrine. It releases the smell and heat from the pit and controls flies. Some households in the study communities used duraplast pipes (100 mm × 6 m long) as vents. In other households, empty insecticide and weedicide containers were fixed together vertically to form a vent. About 47.5% of latrines were observed to have vent pipes. Figure 5 below is a picture of a completed latrine with a vent pipe.

We compared the proportion of households that use latrines for the five different levels of latrine construction (see Table 1). The results show that households at these levels exhibit different habits of latrine use. A Pearson’s Chi-square test indicated that latrine use varied significantly between L1 and L2 (χ^2^ = 73.12, df = 1, *p* < 0.001), L2 and L3 (χ^2^ = 37.76, df = 1, *p* < 0.001), and L3 and L4 (χ^2^ = 96.97, df = 1, *p* < 0.001). 

From Table 1, we observed that latrine use among households experienced two major shifts. The first occurred from L2 to L3 and the second from L3 to L4. At L1 only 5.2% of households switched to latrine use, and at L2, only 13.0%. Therefore, open defecation was still widely practised by households at these levels. At L3, roofing of latrines significantly increased households’ latrine use. Furthermore, the assurance of privacy increased latrine use by 42.5% from L3 (53.1%) to L4 (95.6%). Consequently, we regrouped the latrine construction levels into three levels, as shown in Table 2, based on the probable influencers of latrine use: roofing and privacy. These levels are level-superstructure: households that dug the pit and constructed superstructure, level-roof: households that dug the pit, constructed superstructure and also roofed the latrine, and finally level-privacy: households that dug the pit, constructed superstructure, roofed latrine, and fitted latrine door and vent pipe. Comparing level-superstructure with level-roof, we found that roofing latrines has a significant association with latrine use (χ^2^ = 134.07, df = 1, *p* < 0.001). Households that roofed their latrine use them more than households that only constructed pit and superstructure. Further, a comparison between level-roof and level-privacy revealed that latrine use is higher amongst households whose latrines have doors than those who only roofed their latrines (χ^2^ = 181.75, df = 1, *p* < 0.001).

We found no statistically significant differences in the age, household size, literacy rate, or marital status of respondents and stages of latrine construction (see Table 3). However, the analysis showed that income disparities were associated with the level of latrine construction. Households in level-superstructure have higher average incomes than those in level-roof (F(1,563) = 5.24, *p* = 0.023). Similarly, households in level-privacy have higher average incomes than those in level-roof (F(1,1048) = 4.03, *p* = 0.045). This suggests that households in level-roof have the lowest average income level of the three. 

We also examined the factors likely to influence household’s latrine usage among the levels of latrine completion. We found that 25.4% of respondents at level-superstructure, 12.4% at level-roof and 18.30% at level-privacy reported they would not use their latrines when it is unhygienic. As indicated in Table 4 about 55.3% of latrines at level-privacy were clean compared to 2.8% and 18.8% at level-superstructure and level-roof respectively. Further, we found that about 30% of respondents at each level would not use their latrines when it is damaged. Overall, there was a statistically significant difference in latrine cleanliness among households, with those at level-privacy recording cleaner latrines (F(1,971) = 6.99, *p* = 0.008) than those at level-roof. 

In examining the question of how psychosocial factors influence household latrine completion levels, we present the distribution of our sample across the RANAS factors on latrine construction in Table 5. The statistical analysis revealed that households at level-superstructure perceived a higher risk of contracting diarrhoea than those at level-roof (F(1,564) = 5.630, *p* = 0.018). Latrine construction was also perceived to be significantly more expensive by households at level-superstructure than by those at level-roof (F(1,564) = 5.06, *p* = 0.025). On norms, households at level-superstructure perceived themselves to have significant fewer relatives and fellow community members that constructed their own latrine than those at level-roof (for relatives: F(1,564) = 39.33, *p* = 0.000 for other community members: F(1,564) = 30.20, *p* = 0.000. However, households at level-privacy reported more community members who constructed latrines than those at level-roof; the difference is significant (F(1,1051) = 4.92, *p* = 0.027). The approval of latrine construction by relatives, family members, and friends was higher among respondents at level-privacy than among those at level-roof (F(1,1051) = 4.046, *p* = 0.028). Again, opinion leaders, chiefs, and other community leaders influenced latrine construction by households at level-privacy significantly than those at level-roof (F(1,1051) = 5.57, *p* = 0.018). Beyond that, having the confidence in being able to complete latrine construction even when there are obstructions is significantly higher among respondents at level-privacy than those at level-roof (F(1,1051) = 7.01, *p* = 0.008). However, there was no significant difference between respondents’ commitment level for latrine construction among the levels. 

Respondents gave different reasons for constructing latrines. For instance, 23.7% of the respondents at level-privacy and 10.2% at level-roof stated that they constructed latrines to protect their privacy when defecating (see Table 6). In addition, about 31.6% of respondents at level-privacy, 16.30% at level-roof and 3.6% at level-superstructure said they constructed latrines to improve the health of their household members. Our analysis also showed that, heads of households made the final decisions for latrine construction as reported by majority of respondents (level-superstructure = 77.4%, level-roof = 79.2%, level-privacy = 80.3%). About 24.6% of respondents at level-privacy, compared to 9.3% at level-roof, learned latrine construction by watching other members of their communities. Others (12.2% at level-privacy) learned latrine construction in neighbouring communities whilst some (21.9% at level-roof and 35% at level-privacy) were trained on latrine construction. Among the respondents, 30.9% at level-superstructure, 41.4% at level-roof, and 32.8% at level-privacy planned to collect local raw materials for latrine construction. About 30.2% respondents at level-superstructure, 15.5% at level-roof, and 27.0% at level-privacy were determined to buy materials from the market anytime they ran out of materials when constructing their own latrine. However, about 12.8% of respondents at level-superstructure mentioned they had no plan how to mobilize materials for latrine construction when they ran out of materials, compared to 8.3% and 4.80% at level-roof and level-privacy respectively. Further, about 14.8% of respondents at level-privacy reported they have received subsidies, in the form of building materials, among others, from a Non-Governmental Organisation for latrine construction compared to 2.8% at level-superstructure and 6.3% at level-roof. The community facilitators visited households at level-privacy more often than those at level-roof (F(1,797) = 4.36, *p* = 0.037).

## 4. Discussion 

In this study, we examined factors associated with levels of latrine completion and use in CLTS intervention communities in Northern Ghana. We found that households that roofed their latrines are more likely to switch from open defecation. Households would more likely switch from open defecation to latrine use if latrines protect their privacy. Existing literature on CLTS indicates that convenience, comfort, and health benefits are recurring reasons for latrine use [19,20]. Similarly, a study on rural sanitation showed that privacy and safety are the major reasons for latrine construction and use among women [35]. Other possible reasons related to latrine use among the study households include the cleanliness of the latrine and its structural condition. However, having a latrine did not always translate into latrine use [30]. We observed that about 5.3% (Table 2) of households at level-privacy still practise open defecation. We also found that people are less likely to use latrines when they are dirty and damaged. Latrines that have roof and protect user privacy were also found to be cleaner. These structural characteristics that influence latrine use could be used as benchmarks against which constructed latrines could be measured in CLTS. 

The factors influencing latrine construction and use are broadly classified into demographic and socio-economic factors, knowledge and attitudes, and social influence [13,15,19,20,35,36]. Although we did not find significant differences between household size, age, marital status or literacy rates among respondents across the levels of latrine completion, other research works have reported the influence of these factors on latrine construction and use [36,37,38]. These socio-demographic factors could be further investigated in the study area. Research on economic predictors of latrine ownership in developing countries is not rare. Studies have shown that households with the highest incomes are the most likely to construct latrines [16,39,40]. Household income affects the availability of resources for latrine construction. However, we found in this study that households with the highest incomes were at the lowest level of latrine construction (Table 2). It has also been suggested that, despite the impact of income on latrine construction, other contextual factors drive latrine construction decisions [16,19,20,37]. 

The RANAS factors were used to examine the latrine construction behaviours of households that might have contributed to the construction of latrines in the study area. The perceived risk of diarrhoea was significantly higher for households who constructed only pit and superstructure, while the ownership of a complete latrine as well as latrine use reduced the perceived risk. The perceived risk of a practice has the potential to stimulate and shape households’ behaviours [41,42,43]. However, though risk perception may evoke precautionary measures [44], it is not a determinant of preventive health behaviours [45]. The adoption of healthy behaviours is dependent upon the social process triggered by CLTS [20], social norms [13,19], social networks, and social capital [15,36]. For example, one qualitative study conducted in Benin found prestige to be the main motivation for latrine construction, which had little to do with health risk [46]. In our study, respondents expressed a general feeling of pride in latrine ownership, although those at level-superstructure perceived latrines to be very expensive to construct. CLTS processes produce norms and social networks between members of the community and also with their external environment. Through these social processes, individuals exchange knowledge and ideas and adopt the healthy behaviours of one another whilst strengthening social cohesion and inclusion. In this study, we found that the construction of latrines by relatives and kinsmen has a snowball effect that motivates other community members of the same kin to construct. This result supports previous findings by Shakya et al. [36], which showed that latrine ownership among an individual’s caste is a significant influencer of an individual’s latrine ownership. 

In the study, chiefs, opinion leaders, and family heads played an important role in establishing a norm of latrine construction and use and ending open defecation. Their collective actions brought authority and legitimacy to the whole process of CLTS in their communities and boosted the collective efforts of community members. In both study districts, communities that perceived strong approval of CLTS by their chiefs and opinion leaders had attained ODF status before the endline. Consistent with this finding, a study in Koassanga, Burkina Faso, showed that chiefs and community leaders played a significant role in ensuring collective actions for ending open defecation [15]. In health promotion literature the active involvement of chiefs in the CLTS process enables communities to develop their own context-specific activities to end open defecation and the sustainability of community initiatives on sanitation [15].

Although several researchers and practitioners of CLTS have argued that subsidies may undermine households’ motivation to construct latrines [47,48], our results revealed that subsidies were not a primary driver for latrine construction, however, they helped some households improved the structure and design quality of the latrines. Similarly, a cluster-randomized trial study in Bangladesh by Guiteras, Levinsohn, and Mobarak [49] reported that CLTS latrine construction and latrine use was high among households that received subsidies. In addition to subsidies, monitoring the progress of latrine construction in follow-up visits has been found to have a positive impact on latrine construction and use. Follow-up visits motivate households and provide opportunities to seek technical guidance from field facilitators. Venkataramanan et al. [12] amply acknowledged the influence of follow-up visits on latrine construction and latrine use. 

Looking at the levels of latrine completion separately, households at level-superstructure are at the lowest stage of latrine construction, and open defecation is widely practised among these households. Households at level-superstructure generally expressed higher perceived risk of contracting diarrhoeal disease. They perceived latrine construction to be expensive although they recorded the highest average income. These households have the least clean latrines and would not defecate in their latrines when they are dirty, smelly, or damaged. They have fewer community members and relatives who constructed latrines. In addition, our results suggest that these households had less interaction with field facilitators and also received fewer subsidies than households in other levels of latrine construction. Similarly, a study by Slekiene and Mosler [16] in rural Malawi revealed that households that slowly adopt the innovation of latrine ownership perceived latrine construction to be expensive, communicate less with people about latrines and also feel more vulnerable to contracting diarrheal diseases. Furthermore, Shakya et al. [36] revealed that households social networks significantly influenced their latrine ownership and those with fewer networks are less likely to own latrines. 

Different from households at level-superstructure, those at level-roof recorded the lowest average income and their latrines provide no privacy to users. Therefore, latrine use by households at this level is relatively low compared to level-privacy. Households at level-roof perceived they have more relatives and community members who constructed latrines than did households at level-superstructure. More households at this level reported they received a subsidy than in level-superstructure. 

At level-privacy, households have completed latrines, and open defecation is less practised. Although household latrines at his level are clean, these households would not use latrines when they are dirty or smelly. Households at this level expressed less perceived risk of contracting diarrhoeal diseases but constructed latrines to improve household health and privacy during defecation. They perceived that they have more community members who constructed latrines than do level-roof households, and other important people like family, friends, chiefs and opinion leaders have a significant influence on their latrine construction. These households expressed strong commitment to complete latrine construction even when they encounter challenges. We also observed that level-privacy households had a great deal of interaction with field facilitators and also received more subsidy than households at the other levels.

The sustainability of latrine use is reported to be a great challenge, as it is common for people to abandon full-pit, damaged, or uncompleted latrines and return to open defecation [13,37,50]. This study revealed that households were distributed across different levels of latrine completion and that latrine use varied amongst these levels. Although respondents expressed stronger commitment to complete latrine construction and continued latrine use, continued sensitization is required to sustain their new sanitation behaviour after the departure of project implementers. Scaling-up would therefore require the local planning and development authorities to enhance social marketing campaigns on sanitation to facilitate households to meet their sanitation needs, maintain good sanitation behaviours and create opportunities to act and overcome constraints [46]. 

Some limitations are worth acknowledging, which readers should consider when interpreting the study findings. First, this study used reported family income for the analysis, which does not measure the wealth of households. Respondents may be biased in reporting their real income levels. Second, although we acknowledged the strong relationship between water and sanitation, and households’ health, our research did not discuss this relationship because the whole study was conceptualised on the impact of CLTS intervention on households latrine construction and use. Perhaps, latrine cleanliness may have been influenced by water access and availability. Fourth, all psychosocial determinants were self-reported and measured at endline. Future research should control for multiple comparison errors. Also, as the global community monitors progress on sanitation within the framework of the Sustainable Development Goals, an in-depth understanding of the barriers and complexities associated with sanitation access will inform policy. In this regard, we recommend future research on the relationship between gender, impairment and sanitation access (see [51,52,53]).

## 5. Conclusions

This is the first intervention study to investigate latrine construction behaviours in Ghana and the relation between levels of latrine completion and consequent latrine use. Our results suggest that different factors are associated with households’ latrine completion levels and latrine use. An individual’s decision and action to construct and use a latrine results from the interplay of psychosocial, contextual, and other factors such as the structural and design qualities of the latrine. With the growing interest in scaling up CLTS, this understanding can inform effective strategies for developmental organizations to increase latrine coverage and use and promote the total health of communities. 

## Figures and Tables

**Table 1:** Defecation Habits of Respondents (five levels of latrine construction).

Defecation Habits	L1	L2	L3	L4	L5
Description of levels	only pit	pit + superstructure	pit + superstructure + roof	pit + superstructure + roof + door	pit + superstructure + roof + door + vent pipe
*n*	362	108	96	270	687
Use latrine exclusively	5.2%	13.0%	53.1%	95.6%	94.3%
Defecate in the open exclusively	94.8% ^A,^***	87.0% ^B,^***	46.9% ^C,^***	4.4%	5.7%

^A^ = comparison between L1 and L2. ^B^ = comparison between L2 and L3. ^C^ = comparison between L3 and L4. *** *p* < 0.001.

**Table 2:** Defecation Habits of Respondents (3 levels).

Defecation Habits	Level-Superstructure	Level-Roof	Level-Privacy
Description of Level	(pit + superstructure)	(pit + superstructure + roof)	(pit + superstructure + roof + door + vent pipe)
*n*	470	96	957
Use latrine exclusively	7.0%	53.10%	94.70%
Defecate in the open	93.0% ^A,^***	46.9% ^B,^***	5.3%

^A^ = comparison between Level-superstructure and Level-roof. ^B^ = comparison between Level-roof and Level-privacy. *** *p* < 0.001.

**Table 3:** Socio-demographic Characteristics of Households.

Characteristics of Respondents and Households	Level-Superstructure	Level-Roof	Level-Privacy
*n*	470	96	957
Gender			
*Males*	62.80%	67.70%	66.60%
Age	43.79 (16.17)	44.10 (16.25)	45.89 (15.96)
Household size	9.20 (5.22)	9.93 (4.85)	10.26 (6.41)
Men	3.37 (2.40)	3.74 (2.27)	3.54 (2.22)
Women	3.71 (2.58)	4.41 (3.21)	3.89 (2.80)
children under age 5 years	2.39 (2.13)	2.69 (2.32)	2.54 (2.80)
Income (Ghana Cedis)	164.65 (259.109) ^A,^*	102.37 (125.36) ^B,^*	157.35 (263.92)
Are you able to read and write	17.00%	13.50%	14.40%
Marital Status			
Single married	55.70%	50.00%	51.80%
Single	9.80%	8.30%	6.10%
Widowed	6.60%	8.30%	6.40%
Cohabiting	0.00%	0.00%	0.40%
Divorced/separated	1.10%	0.00%	0.50%
Polygamy married	26.80%	33.30%	34.80%

^A^ = comparison between Level-superstructure and Level-roof. ^B^ = comparison between Level-roof and Level-privacy. * *p* < 0.05. 4.7 Ghana Cedis = 1 US Dollar (exchange rate 26 September 2018).

**Table 4:** Factors that may deter latrine use.

Factors that may Deter Latrine Use	Level-Superstructure	Level-Roof	Level-Privacy
*n*	470	96	957
What are the reasons against the use of your own latrine? ^C^			
Smell	22.20%	19.50%	18.20%
Too dirty/no hygiene	25.40%	12.40%	18.30%
Fear of animals	1.70%	0.00%	0.00%
Fear of falling inside	0.40%	0.00%	0.30%
Queue of people	0.20%	1.00%	0.70%
Latrine is damaged	30.20%	33.30%	32.00%
Full pit	7.60%	7.30%	7.40%
Other reasons	4.00%	9.40%	5.20%
No reason	17.90%	16.70%	16.40%
How clean is your own latrine? ^D^	3.89 (1.13)	4.25 (1.06) ^B,^**	4.56 (0.74)
Cleanliness of latrine (Field observation) ^C^			
Seems unused	14.80%	14.60%	2.60%
Clean: no dirt, no faeces	2.80%	18.80%	55.30%
Somewhat clean. Some dirt but no faeces	1.90%	20.80%	35.70%
Dirty: faeces on slab	0.90%	6.30%	4.70%
Still under construction	79.60%	39.60%	1.70%

^B^ = comparison between Level-roof and Level-privacy, ** *p* < 0.01, ^C^ Multiple response questions are not compared statistically, ^D^ Answer scale: 1 = not clean at all to 5 = very clean.

**Table 5:** Psychosocial Determinants (RANAS) of Latrine Construction.

RANAS Psychosocial Determinants	Level-Superstructure	Level-Roof	Level-Privacy
*n*	470	96	957
**RANAS**	Mean (SD)	Mean (SD)	Mean (SD)
**Risk**			
Generally, how high do you think is the chance/risk that you get diarrhoea?	3.29 (1.41) ^A,^*	2.92 (1.46)	2.89 (1.52)
Imagine that you have diarrhoea, how severe would be the impact on your life?	4.56 (0.83)	4.59 (0.80)	4.57 (0.78)
**Latrine construction attitudes**			
How proud are you of your own latrine?	4.78 (0.59)	4.80 (0.49)	4.79 (0.56)
Do you think you are more respected by your community because you have an own latrine?	4.71 (0.65)	4.56 (0.80)	4.68 (0.69)
If you construct a latrine, do you think you are more vulnerable for envy?	2.55 (1.60)	2.32 (1.45)	2.31 (1.58)
Do you think that constructing your own latrine is expensive?	3.86 (1.23) ^A,^*	3.54 (1.39)	3.58 (1.35)
How difficult is it to find the money to construct your own latrine?	3.99 (1.21)	3.63 (1.28)	3.66 (1.38)
How difficult is it to find the time and effort to construct your own latrine?	2.52 (1.61)	2.84 (1.59)	2.68 (1.58)
**Latrine construction norms**			
When you constructed your latrine, how many members of your community already had one?	2.22 (1.24)	2.30 (1.15)	2.02 (1.20)
How many of your relatives within your community constructed an own latrine?	3.18 (1.57) ^A,^***	4.24 (1.17)	4.35 (1.19)
How many members of your community constructed an own latrine?	3.42 (1.40) ^A,^***	4.25 (1.07) ^B,^*	4.47 (0.92)
How much do people who are important to you (e.g., family, parents, friends) approve that you construct a latrine?	4.43 (0.97)	4.30 (1.13) ^B,^*	4.45 (0.96)
People who are leaders in the community (e.g., opinion leader, Chief of village, etc.) how much do they promote that you construct an own latrine?	4.23 (1.28)	4.22 (1.32) ^B,^**	4.48 (1.00)
How much do you feel a personal obligation to construct an own latrine?	4.52 (0.93)	4.56 (0.87)	4.51 (0.96)
**Abilities**			
**Latrine construction**			
How confident are you that you can construct a latrine even if this is difficult (e.g., gathering the materials)?	4.30 (1.02)	4.50 (0.75)	4.52 (0.77)
How confident are you that you could finish the construction of a latrine even if problems arise (e.g., you run out of money)?	4.12 (1.21)	4.24 (1.16) ^B,^**	4.48 (0.83)
Imagine that the latrine got damaged. How confident are you that you will be able to repair the latrine again?	4.54 (0.84)	4.55 (0.77)	4.66 (0.72)
**Self-Regulation**			
How committed are you to construct your own latrine?	4.65 (0.69)	4.76 (0.45)	4.77 (0.51)

^A^ = comparison between Level-superstructure and Level-roof. ^B^ = comparison between Level-roof and Level-privacy. *** *p* < 0.001, ** *p* < 0.01 and * *p* < 0.05. Answer scale: 1 = not at all to 5 = very much. RANAS: Risk Attitude, Norms, Abilities, Self-Regulation. SD: Standard Deviation.

**Table 6:** Reported reasons for latrine construction.

Reasons for Latrine Construction	Level-Superstructure	Level-Roof	Level-Privacy
*n*	470	96	957
Why did you construct a latrine? ^C^			
Privacy	0.60%	10.20%	23.70%
Security	0.40%	2.10%	5.50%
Health	3.60%	16.30%	31.60%
Hygiene	0.20%	6.20%	15.10%
Comfort	0.20%	1.00%	4.20%
Because of the community meeting done here	0%	0.00%	1.70%
Because some people came and told us to do so	0.40%	6.20%	7.30%
Open defecation is harmful	0.20%	4.20%	8.70%
How did you know how to construct a latrine? ^C^			
Watched other members of the community	0.60%	9.30%	24.60%
Saw it in another community	0.40%	2.00%	12.20%
Someone came from outside and told me	1.90%	21.90%	35%
I just tried without knowledge	0.40%	4.20%	8.80%
Someone else did it for me	1.10%	3.10%	9.60%
My father/husband did the construction	0.40%	7.30%	9.60%
Who in your household made the final decision to construct a toilet? ^C^			
Head of Household	77.40%	79.20%	80.30%
Family all together	13.20%	8.30%	9.30%
Wife	0.60%	2.10%	3.40%
Children	3.60%	1.00%	3.40%
Land lord	1.90%	1.00%	1.00%
Other	3.20%	8.30%	4.80%
Do you have a plan how you can construct a latrine if you are running out of materials? ^C^			
Collect local materials	30.90%	41.40%	32.80%
Buy materials somewhere	30.20%	15.50%	27.00%
Go for local dealers	0.20%	1%	0.60%
Borrow material from others	7.80%	11.50%	11.90%
Ask relatives for help	3.80%	8.30%	5.50%
Sell animals/farm products to buy more materials	12.00%	11.50%	15.20%
Produce the materials by myself	2.30%	1.00%	1.70%
No plan	12.80%	9.40%	4.60%
How much did the construction of your latrine cost in Cedi (GHS)?	338.0 (278.65)	250.18 (277.80)	363.45 (560.36)
Did you receive any subsidy for the construction of your own latrine within the last year?			
Yes	2.80%	6.30%	14.80%
No	97.20%	93.80%	85.20%
What kind of subsidy did you receive? ^C^			
Materials	2.30%	6.30%	12.00%
Labour	0.40%	0.00%	0.90%
Other	0.00%	0.00%	1.80%
Who provided you with this subsidy?			
Government	0.00%	0.00%	0.00%
NGO	2.30%	6.30%	13.70%
People from other communities	0.20%	0.00%	0.50%
Family	0.00%	0.00%	40.00%
Other	0.20%	0.00%	0.10%
How many times did the facilitator follow up in your community	3.02 (1.22)	3.20 (1.02) ^B,^*	3.53 (1.20)

^B^ = comparison between Level-roof and Level-privacy, * *p* < 0.05, ^C^ Multiple response question and was not compared statistically; 4.7 Ghana Cedis (GHS) = 1 US Dollar (exchange rate 26 September 2018). Non-Governmental Organisation (NGO).
